# Positional Correlation Natural Vector: A Novel Method for Genome Comparison

**DOI:** 10.3390/ijms21113859

**Published:** 2020-05-29

**Authors:** Lily He, Rui Dong, Rong Lucy He, Stephen S.-T. Yau

**Affiliations:** 1 Department of Mathematical Sciences, Tsinghua University, Beijing 100084, China; lilyhe6@163.com (L.H.); dongr15@mails.tsinghua.edu.cn (R.D.); 2 Department of Biological Sciences, Chicago State University, Chicago, IL 60628, USA; rhe@csu.edu

**Keywords:** alignment-free, positional correlation natural vector, phylogenetic analysis, genome comparison

## Abstract

Advances in sequencing technology have made large amounts of biological data available. Evolutionary analysis of data such as DNA sequences is highly important in biological studies. As alignment methods are ineffective for analyzing large-scale data due to their inherently high costs, alignment-free methods have recently attracted attention in the field of bioinformatics. In this paper, we introduce a new positional correlation natural vector (PCNV) method that involves converting a DNA sequence into an 18-dimensional numerical feature vector. Using frequency and position correlation to represent the nucleotide distribution, it is possible to obtain a PCNV for a DNA sequence. This new numerical vector design uses six suitable features to characterize the correlation among nucleotide positions in sequences. PCNV is also very easy to compute and can be used for rapid genome comparison. To test our novel method, we performed phylogenetic analysis with several viral and bacterial genome datasets with PCNV. For comparison, an alignment-based method, Bayesian inference, and two alignment-free methods, feature frequency profile and natural vector, were performed using the same datasets. We found that the PCNV technique is fast and accurate when used for phylogenetic analysis and classification of viruses and bacteria.

## 1. Introduction

Predicting the structures, functions, and evolutionary relationships of genes is a fundamental and vital aspect of modern biological research. Therefore, the comparison of genetic sequences is a pivotal step in many protocols and numerous approaches have been employed for this task. Most researchers use conventional alignment-based techniques for sequence comparison; these techniques involve sequence alignment based on selected scoring systems. The algorithms used are generally precise and highlight correlations among sequences. Several sequence alignment methods have been implemented via software packages, such as MrBayes [1]. However, alignment-based methods have disadvantages: they are slow and require a large amount of memory. Furthermore, based on previous studies, multiple sequence alignment (MSA)-based methods cannot be extended with using the huge datasets currently available [2]. Therefore, alignment-free (AF) methods may be used to overcome these problems [3]. Additionally, AF sequence comparison is drawing great interest driven by data-rich applications [4]. A notable common feature of AF approaches is the analysis of special numerical properties of the sequences being compared. High computational efficiency is observed when such techniques are applied to gene and protein data. A series of AF methods for sequence comparison has been developed. AF approaches include iterated-function systems [5], information theory [6], Fourier transformations [7], sequence representations based on chaos theory [8], and moments of the positions of the nucleotides [9,10]. The most widely used AF method is the k-mer-based method and has been published in many excellent journals [11,12,13,14,15,16,17,18,19]. This method involves the analysis of the frequency of strings of specific length k within sequences [20]. Several k-mer-based methods have been developed and applied for the phylogenetic analysis of bacteria and viruses. A notable example is feature frequency profiles (FFP) [21].

Although k-mer-based methods have been applied widely, they do not include positional correlations of nucleotides. However, it is significant to investigate the location for gene sequence comparison. Therefore, positional correlation is important for computational and analytical approaches. Recently, two methods based on moments of the positions of the nucleotides, namely natural vector (NV) [9] and multiple encoding vector (MEV) method [10], were proposed. They were successfully used for the classification and phylogenetic analysis of sequences. The NV method uses frequency, average site, and variance of site to compare sequences. Based on NV, the MEV method can add information about the chemical and physical properties of a nucleotide. The distribution of four bases is considered independently in these two methods. However, it has been reported that the four bases are correlated; in fact, the correlation of nucleotides is based on the widely applied hidden Markov model (HMM) [22]. In the present study, we propose a novel 18-dimensional numerical feature vector method to characterize DNA sequences. The method is named positional correlation natural vector (PCNV) to characterize DNA sequences. Our vector contains the frequency, average, and variance the locations of four bases. Furthermore, we added the position correlation of each pair of the four bases as important features. We tested the PCNV method using several datasets and compared it with the alignment-based Bayesian inference approach, which can be applied using MrBayes software [1], as well as two AF methods, FFP and NV.

## 2. Results

To demonstrate that PCNV is effective, we applied it to different datasets: the genomes of hepatitis C virus (HCV), hepatitis B virus (HBV), human papillomavirus (HPV), dengue virus (DENV), and 59 bacterial species. The length of the sequences studied ranged from thousands to millions of base pairs. For each dataset, the PCNVs of the sequences were computed using MATLAB R2016a and phylogenetic trees were reconstructed using MEGA 7. Finally, we evaluated the performance of our methods based on sensitivity, specificity, and accuracy. Computations were performed on a PC with Intel Core i7-6560U CPU @ 2.20 GHz and 8 GB RAM.

### 2.1. Phylogeny of HCV

**Figure 1:**
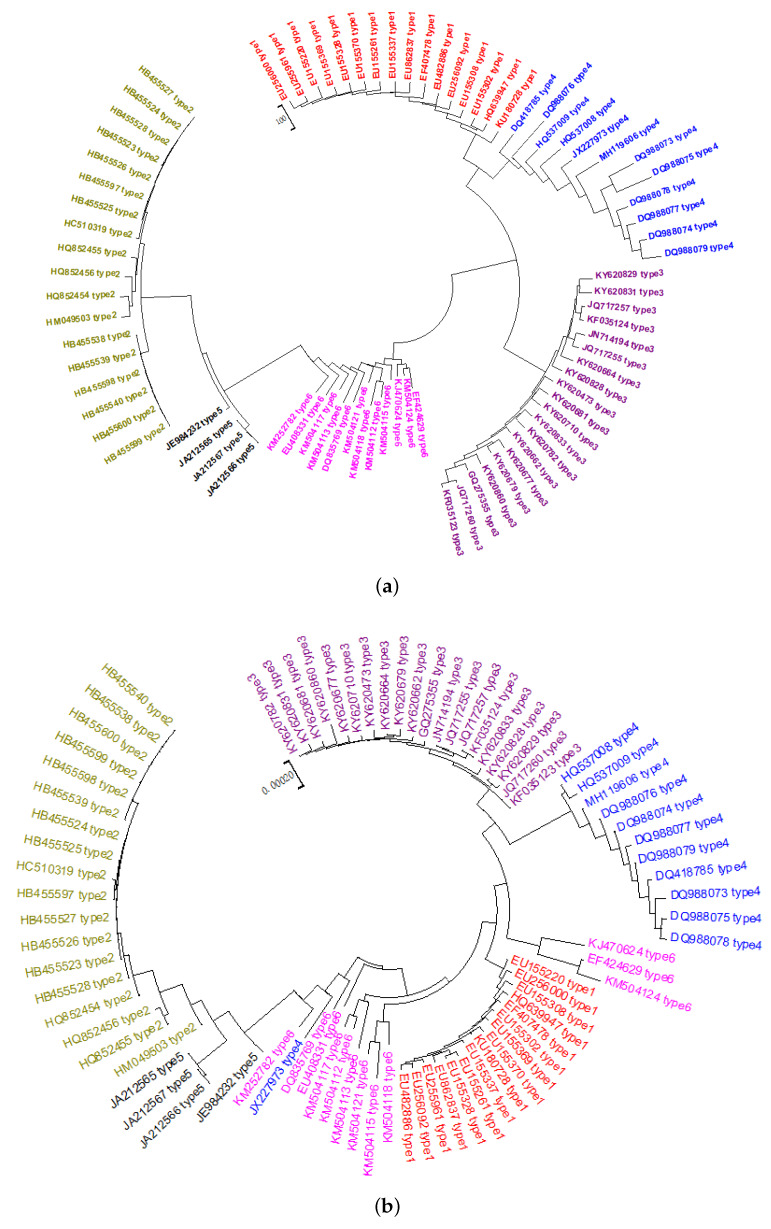

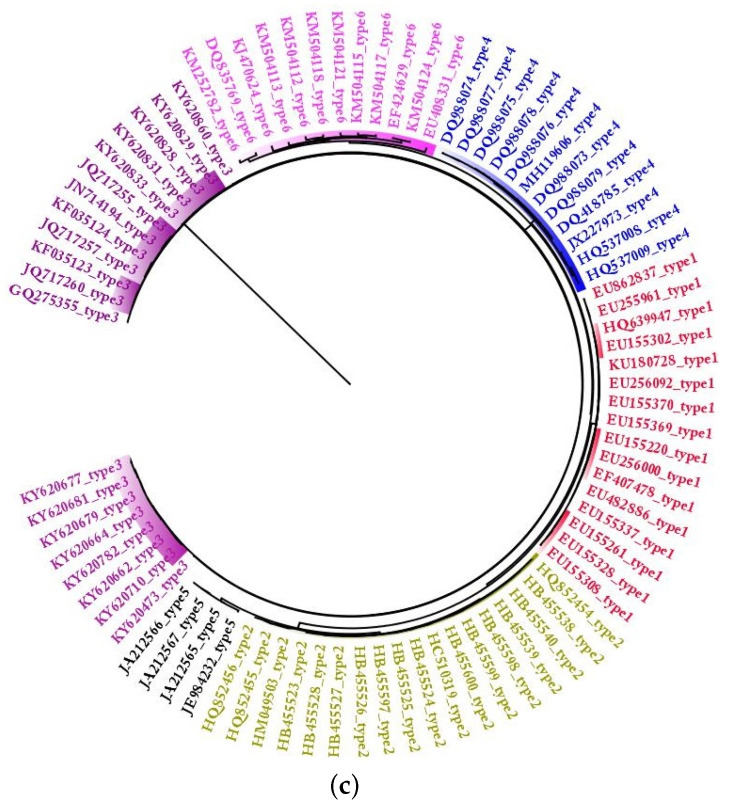
(**a**) The Neighbor-Joining phylogenetic tree of 82 HCV genome sequences based on PCNV method. (**b**) The Neighbor-Joining phylogenetic tree of 82 HCV genome sequences based on FFP method (k = 6). (**c**) The phylogenetic tree of 82 HCV genome sequences based on Bayesian inference method.

Using our PCNV method, 82 HCVs are correctly clustered into six clades, as shown in Figure 1a [23]. Using the FFP method, the value of k for analysis of these viruses is 6. As shown in Figure 1b, some Genotype 6 and 1 HCVs are clustered together incorrectly in the FFP phylogenetic tree. Furthermore, a sequence in Genotype 4 is assigned to Genotype 6. PCNV produces better results for this dataset than FFP. The Bayesian inference method was also utilized for the evolutionary analysis of this dataset. Figure 1c shows that this method divides Genotype 3, shown in violet, into two groups.

### 2.2. Phylogeny of HBV

**Figure 2:**
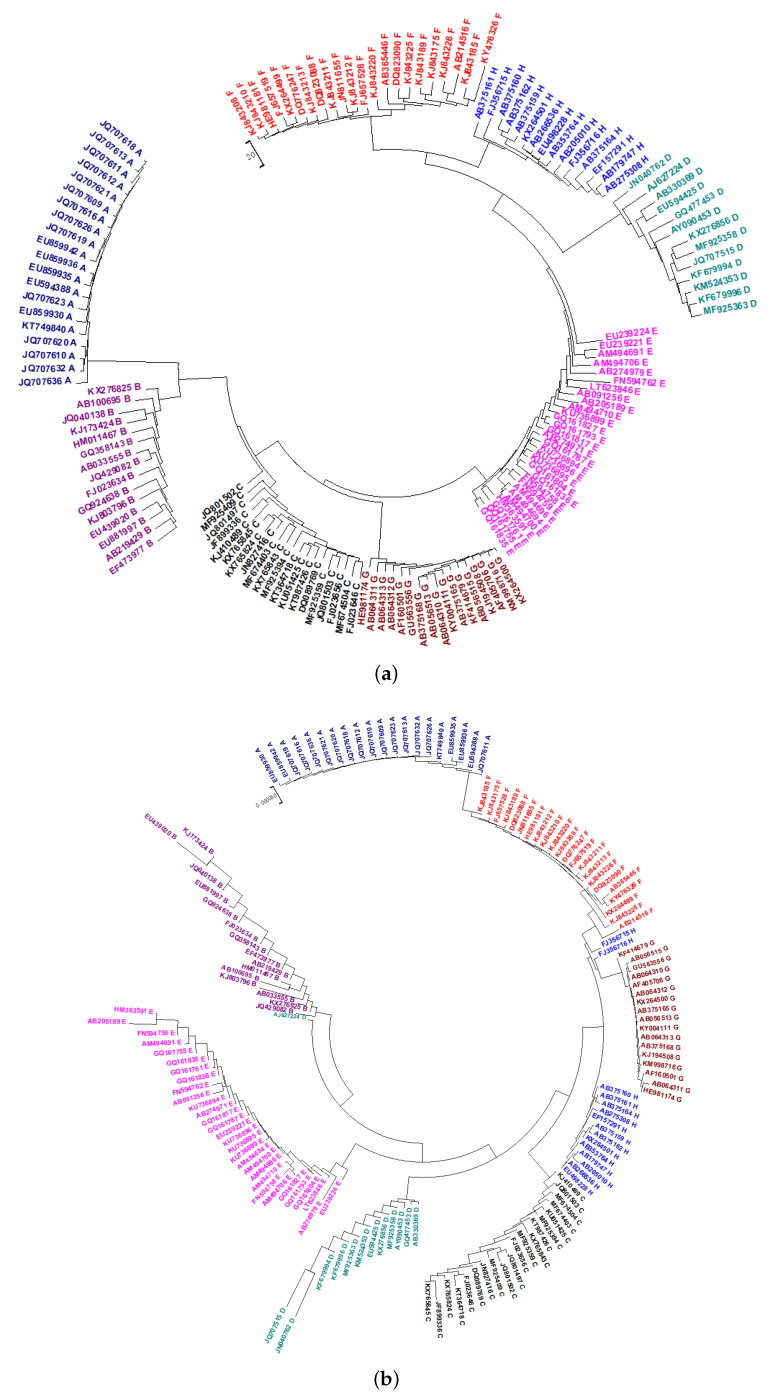

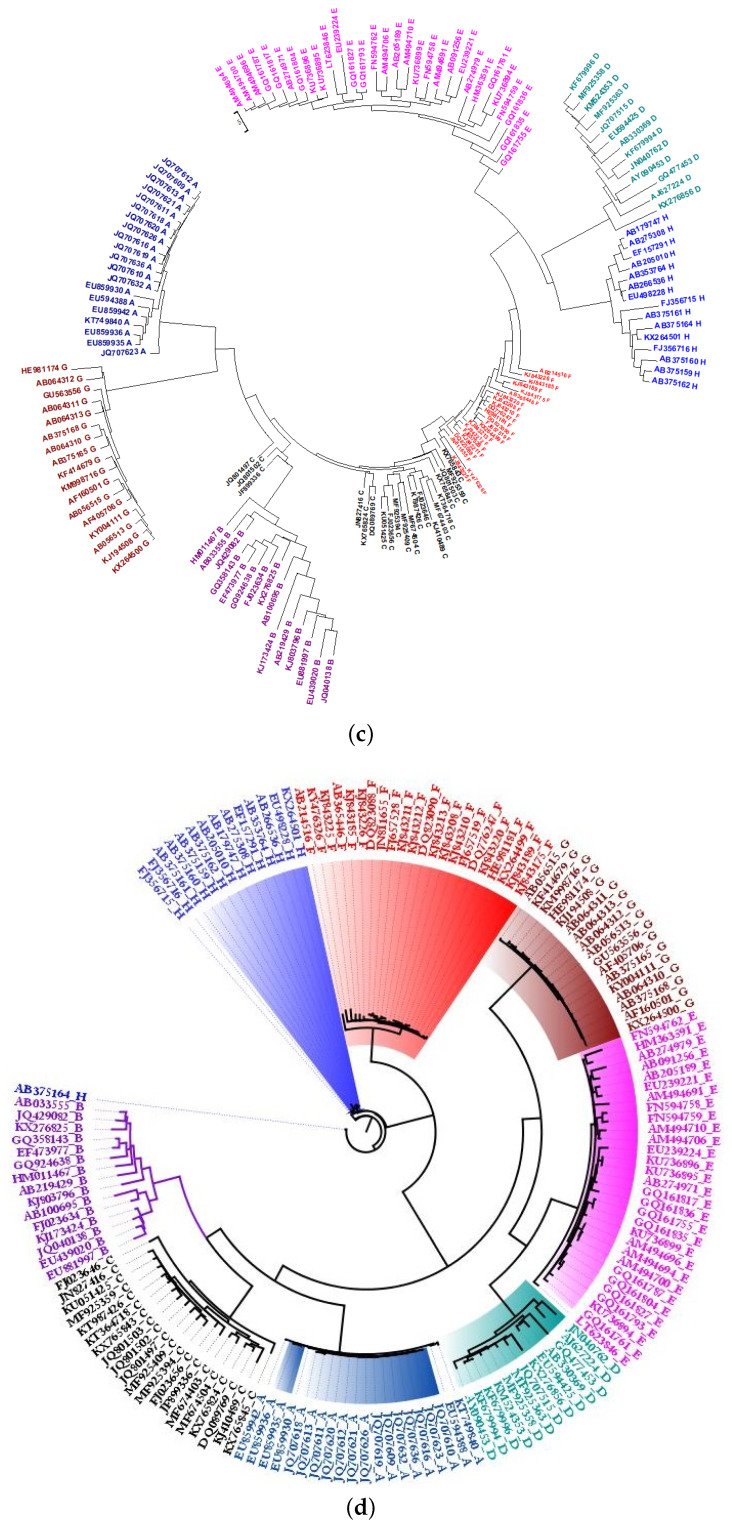
(**a**) The Neighbor-Joining phylogenetic tree of 152 HBV genome sequences based on PCNV method. (**b**) The Neighbor-Joining phylogenetic tree of 152 HBV genome sequences based on FFP method (k = 5). (**c**) The Neighbor-Joining phylogenetic tree of 152 HBV genome sequences based on NV method. (**d**) The phylogenetic tree of 152 HBV genome sequences based on Bayesian inference method.

Using PCNV, 152 HBVs are correctly divided into eight lineages, as shown in Figure 2a. The phylogenetic tree created using the FFP method is shown in Figure 2b. According to the HBV database, “AJ627224” belongs to Genotype D. However, according to the FFP method, it is related to Genotype B. The FFP method also cluster “FJ356715” and “FJ356716”, belonging to Genotype H, to Genotypes F and G, respectively. Therefore, for this dataset, our PCNV method is superior to FFP. The phylogenetic tree created using the traditional NV method is shown in Figure 2c. However, in this tree, three Genotype C viruses are classified into other groups. The phylogenetic tree created using Bayesian inference is shown in Figure 2d. It shows that “AB371164” belongs to Genotype H, as separated from Genotype H. This is an indication that the positional correlation between nucleotides can improve the accuracy of classification.

### 2.3. Phylogeny of DENV

**Figure 3:**
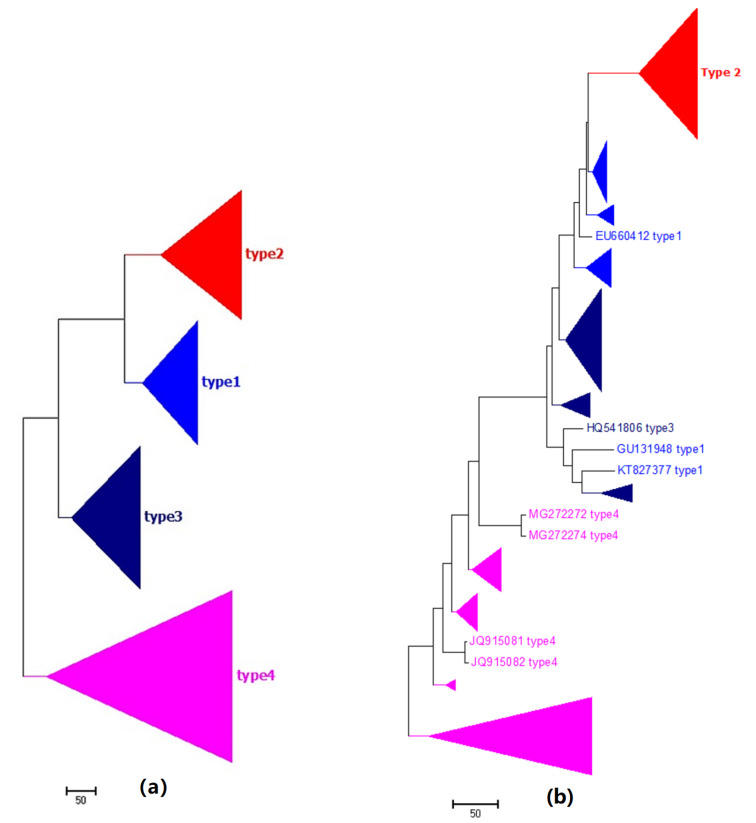
(**a**) The Neighbor-Joining phylogenetic tree of 330 dengue viruses genome sequences based on PCNV method. (**b**) The Neighbor-Joining phylogenetic tree of 330 dengue viruses genome sequences based on NV method.

As shown in Figure 3a, the phylogenetic tree constructed using PCNV classifies all viruses into the correct categories. However, as shown in Figure 3b, the NV method divides Genotype 1 into two clusters. Therefore, once again, it is clear that positional correlation between nucleotides can effectively improve the NV method.

### 2.4. Phylogeny of HPV

**Figure 4:**
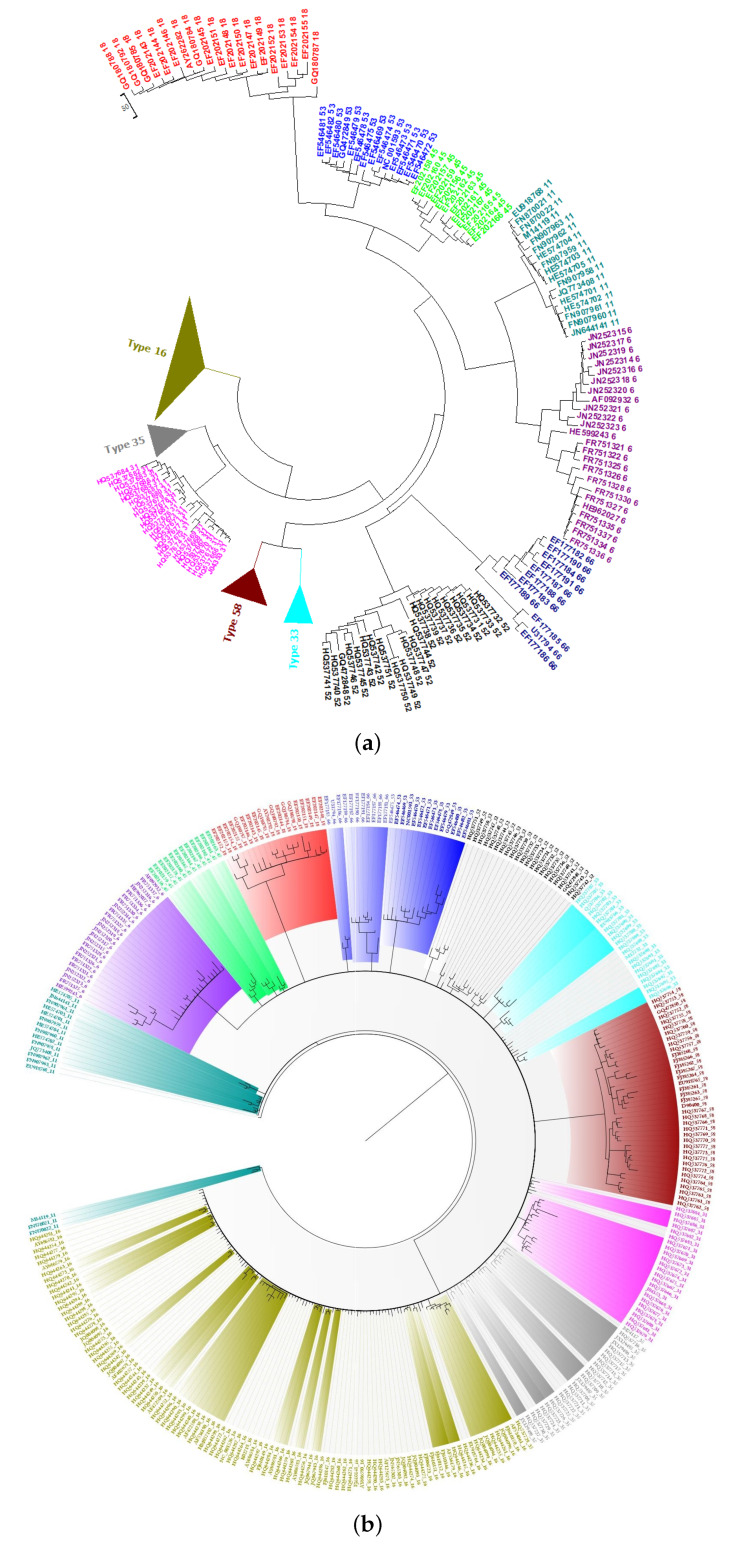
(**a**) The Neighbor-Joining phylogenetic tree of 326 HPV genome sequences based on PCNV method. (**b**) The phylogenetic tree of 326 HPV genome sequences based on Bayesian inference method.

We found that PCNV categorizes the dataset into the correct biological groups in 0.78 s (Figure 4a; Table 1); this is much faster than the FFP method, which takes 35 s (Table 1). The Bayesian inference method divide Genotype 11 into two parts, as highlighted in cyan in Figure 4b.

### 2.5. Phylogeny of Bacteria

**Figure 5:**
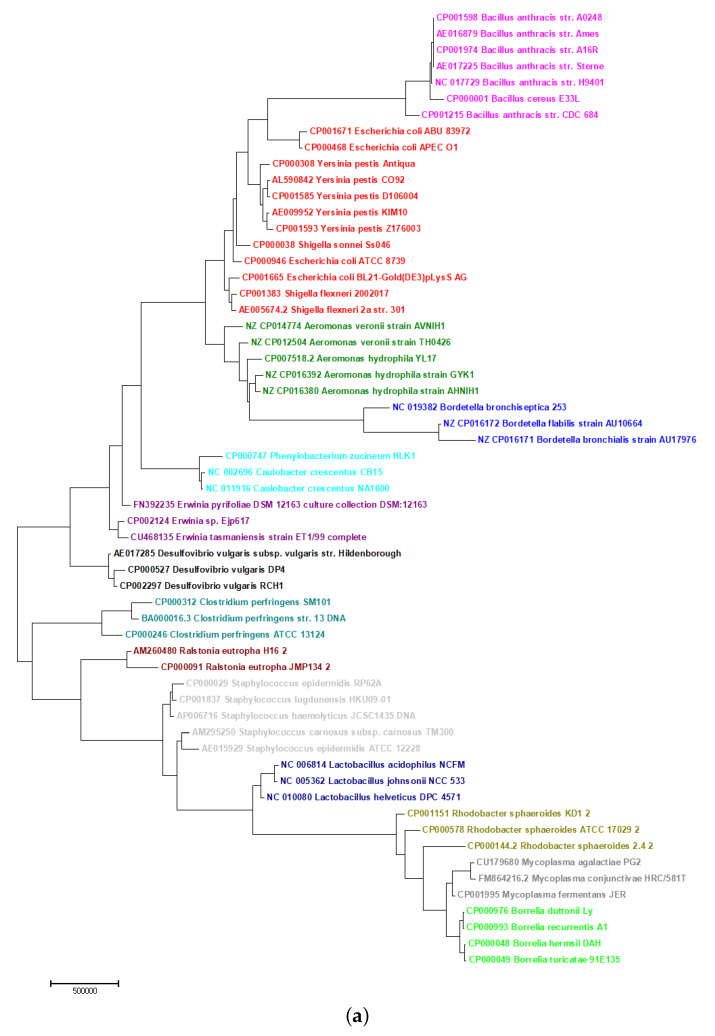

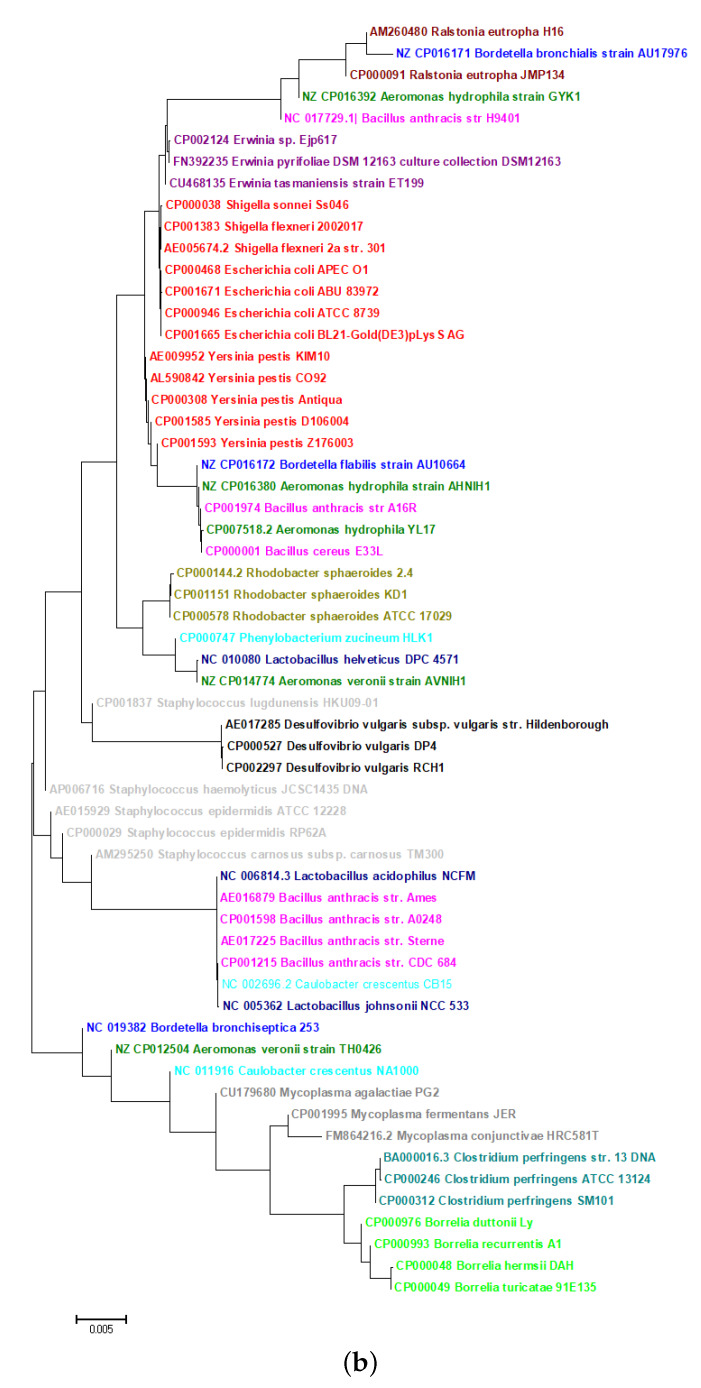
(**a**) The Neighbor-Joining phylogenetic tree of 59 bacteria genome sequences based on PCNV method. (**b**) The Neighbor-Joining phylogenetic tree of 59 bacteria genome sequences based on FFP method (k = 11).

The dataset consisted of 14 families, as shown in Figure 5a,b, of bacterial species with long genomes that ranged from 0.8 to 5 million bp. Using the PCNV method, the phylogenetic tree of these organisms was reconstructed. As shown in Figure 5a, the 59 bacterial species are divided into 14 families that are separated from each other. The 11-mer FFP method mixed these families (Figure 5b). Additionally, the run time for FFP is more than a day, which is far longer than the time required for PCNV. Bayesian inference takes even longer, to the extent that it is not possible to complete the analysis using this method in Muscle on a server equipped with an Intel Xeon E5-2667 v3 Processor and Linux Home Premium with 384 GB RAM (Table 1).

### 2.6. Classification

Besides evolutionary analysis, PCNV can also be used for classification. Both FFP and Alignment-Free-Kmer-Statistics (AFKS) [16], based on the k-mer approach, can also be used for classification. However, the question of how to choose the value of k is not easily answered. In the present paper, for the FFP method, we set the k value as the minimum integer of $log_{4}\left(N\right)$, i.e., $k=floor(log_{4}\left(N\right))$, where N is the maximum length of the sequences studied [21]. For the AFKS method, we used $k=floor(log_{4}\left(\frac{1}{n}\sum_{i\in S}len\left(i\right)\right))$, where n is the number of sequences in the set S [16].

In the PCNV method, after computing the distance matrix using each approach, the one-nearest neighbor (1-NN) [24] method was used for predictions. The sensitivity, specificity, and accuracy of the predictions made using each method are shown in Table 2. It is clear that PCNV is superior to the other two algorithms in this study.

## 3. Discussion

In the present paper, we propose a novel 18-dimensional vector method for genome comparison. This PCNV method can be used to successfully define the distribution of nucleotides based on information on the frequency and position of DNA sequences. The correlation of position between two different bases is used in addition to the average position and variance of position of each base. As a result, a high-dimensional DNA genome sequence is reduced to an 18-dimensional numerical vector. Correlations in base distribution play a key role in sequence comparison. Usually, conventional alignment-based methods produce reasonable phylogenetic trees and are therefore widely applied. However, when the dataset volume is large or the sequences analyzed are very long, these methods become ineffective. The phylogenetic analysis results on several distinct datasets show that PCNV can quickly and accurately compare massive datasets of long DNA sequences. We also compared our method with three methods: the popular alignment-based Bayesian inference method, the alignment-free FFP, and Natural Vector methods. The results show that our method can construct more accurate evolutionary relationships among sequences.

To demonstrate the computational advantage of PCNV, we compared our running time constructing phylogenetic trees with FFP, AFKS, Bayesian inference, and Muscle. Compared with the two extensively applied alignment-free methods FFP and AFKS, the running time of PCNV is smallest for all datasets and even takes less than 1 s, except on the bacteria dataset. For bacteria dataset, PCNV is extremely fast and only takes about 53 s, while FFP and AFKS take more than one day. Compared with the two alignment-based methods Bayesian inference and Muscle, PCNV takes much less time for all datasets. For bacteria dataset, Bayesian inference and Muscle cannot obtain phylogeny tree within several days.

With long DNA sequences, particularly bacterial genomes, the Bayesian inference method was much slower than the PCNV approach, sometimes to the extent that it simply did not work, the main reason being that Bayesian inference method is based on alignment method, such as Muscle (Table 1). Even alignment-free methods such as FFP and AFKS were slow in comparison with PCNV, especially when the analyzed sequences were longer, as shown in Table 1.

Furthermore, the MEV was used for comparison. Although both PCNV and MEDV studies aim to solve the problem of genetic sequence comparison, they have significant differences in the features extracted from the sequences. MEV method did not consider the position correlation feature which is an important source of information for genetic sequences. The novelty of our new method is that it designs six suitable features to characterize the correlation among nucleotide position in sequences. The second difference is that our new method does not categorize four types of nucleotides into three groups according to their chemical properties. The third difference is that our new method applies the popular neighbor-joining algorithm to construct phylogenetic trees, while the old study used the UPGMA algorithm which may produce misleading trees.

To show the advantages of our new method (PCNV), MEV and PCNV were compared using all datasets studied used in for the present study. The Neighbor-Joining (NJ) algorithm is used in tree construction of both methods. The NJ trees built by the MEV method are shown in Figures S1–S5. As shown in Figure S1, there are six types of HCV dataset. Using the MEV method, the type 6 marked in pink is divided into two parts. In the NJ tree of the HBV dataset shown in Figure S2, the virus KX765843 belonging to clade C (marked in black) is incorrectly assigned to clade F (marked in red). Similarly, as shown in Figure S3, types 1 (marked in blue) and 3 (marked in navy) of dengue are mixed together. In Figure S4, two viruses from type 35 group (marked in gray) are categorized into other groups.

Horizontal (or lateral) gene transfer (HGT) is a common phenomenon in bacteria. Due to the problem of HGT, Koski and Golding [25] even stated that genes appearing to be the most similar based on BLAST hits are often not the closest relatives each other phylogenetically. It means when there are HGT, if alignment is used, there may be mistakes. For example, given two distantly related bacteria that have exchanged a gene, a phylogenetic tree including those species will show them to be closely related because that gene is the same, even though most other genes are dissimilar. For bacteria, due to the extensively existing HGT, the phylogenetic tree based on alignment may be misleading. To get correct phylogenetic trees of bacteria, one main method is the 16s rRNA-based method, which constructs trees according to the alignment of 16s ribosomal RNA, i.e., 16s rRNA. The 16s rRNA gene tends to be conserved among bacteria with close phylogenetic distances, and thus is often not affected by HGT, but has enough variable differences. However, the method of 16sRNA loses some information in the whole genome. Our PCNV method uses the whole genome sequence and needs no sequence alignment, thus has the potential to be not affected by the HGT and obtain the correct phylogenetic relationship among bacteria.

**Figure 6:**
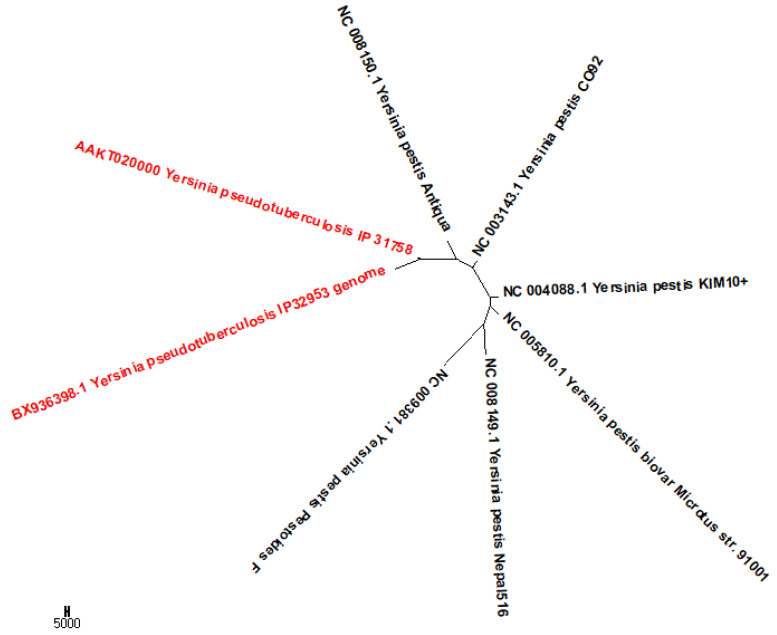
The Neighbor-Joining phylogenetic tree of eight Yersinia genomes based on PCNV method.

To show that our method may be not affected by HGT, we used another dataset of eight Yersinia genomes in a previous study [4]. The eight Yersinia genomes are too similar in sequence for classical phylogenetic inference, but share gene segments. The dataset includes two *Yersinia pseudotuberculosis* and six *Yersinia pestis* complete genomes. Using PCNV, we get the neighbor-joining tree of the eight bacteria shown in Figure 6. For the figure, we see that the two *Yersinia pseudotuberculosis* isolates form sisters and are separate from the six *Yersinia pestis* genomes.

Genetic distance is a measure of the genetic divergence between species or between populations within a species, whether the distance measures time from common ancestor or degree of differentiation. Several genetic distances have been proposed based on different evolutionary models. The genetic distance can only be applied to alignment results. A commonly used measure of genetic distance is the fixation index, which varies between 0 and 1. Our PCNA is an alignment-free approach; we measure the distance between species using Euclidean distance and we do not assume any evolutionary model. This distance is positively correlated with their genetic distance, since it can successfully measure the divergence between two species as well. In the neighbor-joining trees constructed by PCNV, sum of the length of the branches traversed from one species to another is equal to their distance and thus positively correlated with their genetic distance. Due to difference in mutation rates for species, for two given datasets, the average internal genetic distance in each dataset may be different enough. In this case, the average distance obtained with PCNV for two datasets may have a big difference, which leads to very different scales in the two derived phylogenetic trees. For the same dataset, different alignment-free method may produce different average distance and thus produce different scales of phylogenetic trees.

Our new method has several limitations, for example we cannot work out the time of evolution. These limitations will need to be studied in the future.

## 4. Materials and Methods

### 4.1. Dataset

Five datasets were used to test and verify the new technique.

#### 4.1.1. HCV

Hepatitis C is a liver infection caused by HCV. The World Health Organization (WHO) estimates that HCV infects 3% of the world’s population [26]. Because this virus causes few symptoms, diagnosis is difficult in many cases [27]. In the present study, we acquired 82 complete HCV genomes from the Virus Pathogen Database and Analysis Resource (ViPR) [23]. This dataset has a genomic length of 8957–9666 nucleotides. The NCBI accession numbers are shown in Table S1.

#### 4.1.2. HBV

HBV is a hepatotropic virus that can establish a persistent and chronic infection in humans through immune anergy. It exhibits formidable morbidity and mortality in humans and currently infects 3.5% of the global population [28,29]. HBV is genetically diverse and comprises 10 different genotypes, designated A–J [29,30]. Additional subgenotypes exist within Genotypes A–D and F [31]. The HBV genotypes differ in their geographic distributions. Identifying HBV genotypes quickly and accurately is very important for clinical diagnosis. In the present work, 152 complete HBV genomes including eight genotypes (A–H), were downloaded from the Hepatitis B Virus Database (HBVdb) [30]. The NCBI accession numbers are shown in Table S2.

#### 4.1.3. DENV

DENVs are mosquito-borne aviviruses that have plagued humans for centuries [32]. Statistics show that DENVs infect up to 390 million people worldwide each year; 25% of these people suffer from clinical disease. With four antigenically distinct but immunologically cross-reactive serotypes (DENV-1–4), dengue has one of the most complex transmission processes of all infectious diseases in human populations [33]. Therefore, it is important to distinguish which group particular DENVs belong to. In the present study, 330 dengue viruses were used to demonstrate the effectiveness of the method. The NCBI accession numbers are shown in Table S3.

#### 4.1.4. HPV

HPV is a circular double-stranded DNA virus that causes a variety of proliferative epithelial lesions at specific anatomical sites. It is also the most common sexually transmitted virus. There are many different types of HPV, several of which cause health problems such as genital warts and cancer [34]. For example, the low-risk HPV Types 6 and 11 can cause genital warts or benign cell changes, while the high-risk HPV Types 16 and 18 cause about 70% of cervical cancers [35]. Therefore, identification of HPV genotypes in infected patients is particularly important. In the present work, 326 complete genomes of 12 HPV strands (6, 11, 16, 18, 31,33, 35, 45, 52, 53, 58, and 66) were studied. All viral genomes in this HPV dataset are publicly available at GenBank or NCBI databases. The NCBI accession numbers are shown in Table S4.

#### 4.1.5. Bacteria

There is a biomass of bacteria, the main representatives of the prokaryotes, on Earth. Researchers usually investigate evolutionary relationships among bacteria by building phylogenetic trees. Owing to the large genome size (>1 million bp) of bacteria, it is impossible to reconstruct a bacterial phylogenetic tree in a reasonable amount of time using traditional multiple sequence alignment methods with current computational technology. We used 59 bacterial species to test our method (Table 3). The NCBI accession numbers are shown in Table S5.

### 4.2. Positional Distribution

Let $S=s_{1},s_{2},...,s_{N}$ be a DNA sequence of length *N*. Denote $n_{\alpha }$ as the number of nucleotides α in the sequence, where α∈{A,C,G,T}. Here, $p_{\alpha_{j}}$ is the position of the nucleotide α at the *j*th appearance, $j=1,2,\cdots ,n_{\alpha }$, and $p_{\alpha_{0}}=0$. Obviously, $p_{\alpha_{j}}<p_{\alpha_{j+1}}\left(0\leq j<n_{\alpha }\right)$. For example, given the sequence “ACTGGCAAT”, $n_{A}=3,\;p_{A_{1}}=1,\;p_{A_{2}}=7,\;p_{A_{3}}=8$. We first define the positional distribution of α ($U_{\alpha }\left(i\right),i=1,2,\cdots ,N$) as follows:

(1) $$\begin{array}{c} U_{\alpha }\left(i\right)=\left\{\begin{array}{c} \frac{p_{\alpha_{j}}}{p_{\alpha_{j+1}}-p_{\alpha_{j}}};p_{\alpha_{j}}\leq i<p_{\alpha_{j+1}};j=0,1,\cdots ,n_{\alpha }-1.\\ \frac{p_{\alpha_{n_{\alpha }}}}{N-(p_{\alpha_{n_{\alpha }}}-1)};\;p_{\alpha_{n_{\alpha }}}\leq i\leq N.\;\;\;\; \end{array}\right. \end{array}$$

The positional distribution of α(α∈A,C,G,T) is

(2) $$\begin{array}{c} \underset{p_{\alpha_{1}}-1}{\underset{︸}{0,0,\cdots ,0}},\underset{p_{\alpha_{2}}-p_{\alpha_{1}}}{\underset{︸}{\frac{p_{\alpha_{1}}}{p_{\alpha_{2}}-p_{\alpha_{1}}},\frac{p_{\alpha_{1}}}{p_{\alpha_{2}}-p_{\alpha_{1}}},\cdots ,\frac{p_{\alpha_{1}}}{p_{\alpha_{2}}-p_{\alpha_{1}}}}},\cdots ,\\ \underset{p_{\alpha_{n_{\alpha }}}-p_{\alpha_{n_{\alpha }-1}}}{\underset{︸}{\frac{p_{\alpha_{n_{\alpha }-1}}}{p_{\alpha_{n_{\alpha }}}-p_{\alpha_{n_{\alpha }-1}}},\frac{p_{\alpha_{n_{\alpha }-1}}}{p_{\alpha_{n_{\alpha }}}-p_{\alpha_{n_{\alpha }-1}}},\cdots ,\frac{p_{\alpha_{n_{\alpha }-1}}}{p_{\alpha_{n_{\alpha }}}-p_{\alpha_{n_{\alpha }-1}}}}},\;\\ \underset{N-(p_{\alpha_{n_{\alpha }}}-1)}{\underset{︸}{\frac{p_{\alpha_{n_{\alpha }}}}{N-(p_{\alpha_{n_{\alpha }}}-1)},\frac{p_{\alpha_{n_{\alpha }}}}{N-(p_{\alpha_{n_{\alpha }}}-1)},\cdots ,\frac{p_{\alpha_{n_{\alpha }}}}{N-(p_{\alpha_{n_{\alpha }}}-1)}}} \end{array}$$

Example: Given the sequence “ACTGGCAAT”, the positional distribution is as shown in Table 4. Here, we take α=C as an example to show the details of the calculation process. According to the above definition: $N=9,\;n_{C}=2;\;P_{C_{0}}=0,\;P_{C_{1}}=2,\;P_{C_{2}}=6.$

(1) When $p_{C_{0}}\leq i<p_{C_{1}}$, namely, i=1, so $U_{C}\left(1\right)=\frac{p_{C_{0}}}{p_{C_{1}}-p_{C_{0}}}=0$.
(2) When $p_{C_{1}}\leq i<p_{C_{2}}$, namely, i∈{2,3,4,5}, thus $U_{C}\left(2\right)=U_{C}\left(3\right)=U_{C}\left(4\right)=U_{C}\left(5\right)=\frac{p_{C_{1}}}{p_{C_{2}}-p_{C_{1}}}=\frac{2}{6-2}=\frac{2}{4}$.
(3) When $p_{C_{2}}\leq i\leq N$, namely, i∈{6,7,8,9}, $U_{C}\left(6\right)=U_{C}\left(7\right)=U_{C}\left(8\right)=U_{C}\left(9\right)=\frac{p_{C_{2}}}{N-(p_{C_{2}}-1)}=\frac{6}{9-(6-1)}=\frac{6}{4}$.

Similarly, we can get $U_{A},U_{G},U_{T}.$

### 4.3. Position Correlation Vector

#### 4.3.1. Average Positional Distribution

The average positional distribution $\kappa_{\alpha }$ is defined as (α,β∈{A,C,G,T}):

(3) $$\kappa_{\alpha }=\frac{\sum_{i=1}^{N}U_{\alpha }\left(i\right)}{n_{\alpha }},\;\alpha \in \left\{A,C,G,T\right\}.$$

Therefore,

(4) $$\begin{array}{c} \kappa_{\alpha }=\frac{\sum_{i=1}^{N}U_{\alpha }\left(i\right)}{n_{\alpha }}=\frac{\sum_{j=0}^{n_{\alpha }-1}\frac{p_{\alpha_{i}}}{p_{\alpha_{j+1}}-p_{\alpha_{j}}}\times \left(p_{\alpha_{j+1}}-p_{\alpha_{j}}\right)}{n_{\alpha }}\\ \;+\frac{\frac{p_{n_{\alpha }}}{N-(p_{n_{\alpha }}-1)}\times \left[N-\left(p_{n_{\alpha }}-1\right)\right]}{n_{\alpha }}=\frac{\sum_{j=0}^{n_{\alpha }}p_{\alpha_{j}}}{n_{\alpha }}. \end{array}$$

For the example sequence “ACTGGCAAT” above, $\kappa_{A}=\frac{\sum_{i=1}^{N}U_{A}\left(i\right)}{n_{A}}=\frac{\frac{1}{6}\times 6+\frac{7}{1}+\frac{8}{2}\times 2}{3}=\frac{\frac{\sum_{j=0}^{n_{A}}p_{A_{j}}}{n_{A}}}{3}=\frac{1+7+8}{3}=\frac{16}{3}$, and likewise for C, G, and T.

#### 4.3.2. Positional Covariance

The positional covariance of nucleotide α and β (cov(α,β)) are defined as follows:

(5) $$cov\left(\alpha ,\beta \right)=\sum_{i=1}^{N}\frac{\left(U_{\alpha }\left(i\right)-{\bar{U}}_{\alpha }\right)\left(U_{\beta }\left(i\right)-{\bar{U}}_{\beta }\right)}{n_{\alpha }\cdot n_{\beta }}.$$

where

(6) $${\bar{U}}_{\alpha }=\frac{1}{N}\sum_{i=1}^{N}U_{\alpha }\left(i\right)=\frac{1}{N}\sum_{j=0}^{n_{\alpha }}p_{\alpha_{j}}.$$

For the sequence “ACTGGCAAT”, the positional distributions of A and C are $\left(\frac{1}{6},\frac{1}{6},\frac{1}{6},\frac{1}{6},\frac{1}{6},\frac{1}{6},7,\frac{8}{2},\frac{8}{2}\right)$ and $\left(0,\frac{2}{4},\frac{2}{4},\frac{2}{4},\frac{2}{4},\frac{6}{4},\frac{6}{4},\frac{6}{4},\frac{6}{4}\right)$, respectively.

${\bar{U}}_{A}=\frac{1}{N}\sum_{i=1}^{N}U_{A}\left(i\right)=\frac{\sum_{j=0}^{n_{A}}p_{A}}{N}=\frac{\frac{1}{6}\times 6+\frac{7}{1}+\frac{8}{2}\times 2}{9}=\frac{16}{9}$. 

${\bar{U}}_{C}=\frac{1}{N}\sum_{i=1}^{N}U_{C}\left(i\right)=\frac{\sum_{j=0}^{n_{C}}p_{C}}{N}=\frac{0+\frac{2}{4}\times 4+\frac{6}{4}\times 4}{9}=\frac{8}{9}$.

$cov\left(A,C\right)=\sum_{i=1}^{N}\frac{\left(U_{A}\left(i\right)-{\bar{U}}_{A}\right)\left(U_{C}\left(i\right)-{\bar{U}}_{C}\right)}{n_{A}\cdot n_{C}}=\frac{1}{3*2}[\left(\frac{1}{6}-\frac{16}{9}\right)\left(0-\frac{8}{9}\right)+\left(\frac{1}{6}-\frac{16}{9}\right)\left(\frac{2}{4}-\frac{8}{9}\right)\times 4+\left(\frac{1}{6}-\frac{16}{9}\right)\left(\frac{6}{4}-\frac{8}{9}\right)\;+\left(\frac{7}{1}-\frac{16}{9}\right)\left(\frac{6}{4}-\frac{8}{9}\right)+\left(\frac{8}{2}-\frac{16}{9}\right)\left(\frac{6}{4}-\frac{8}{9}\right)\times 2]=1.4769$.

The same method is used to compute cov (A;G); cov (A; T); cov (C;G); cov (C; T); and cov (G; T).

#### 4.3.3. Positional variance

The positional variance $D_{\alpha }$ is described as:

(7) $$D_{2}^{\alpha }=cov\left(\alpha ,\alpha \right)=\sum_{i=1}^{N}\frac{\left(U_{\alpha }\left(i\right)-{\bar{U}}_{\alpha }\right)\left(U_{\alpha }\left(i\right)-{\bar{U}}_{\alpha }\right)}{n_{\alpha }\cdot n_{\alpha }}.$$

Consequently, an 18-dimensional PCNV of a DNA sequence was constructed as follows:

$$(n_{A},n_{C},n_{G},n_{T},\kappa_{A},\kappa_{C},\kappa_{G},\kappa_{T},D_{2}^{A},D_{2}^{C},D_{2}^{G},D_{2}^{T},cov\left(A,C\right),cov\left(A,G\right),cov\left(A,T\right),cov\left(C,G\right),\;cov(C,T),cov(G,T)).$$

Then, we used the Euclidean distance to compute the pairwise distance among the 18-dimensional vectors of the genome sequences. A phylogenetic tree can be built using the NJ algorithm in MEGA 7.0 software [36].

Additionally, the PCNV method can be used to classify organisms. Naturally, sensitivity, specificity, and accuracy were used to evaluate classification performance. The definitions of these measures are as follows:

(8) Sensitivity=TP/(TP+FN).

and

(9) Specificity=TN/(FP+TN).

where TP, TN, FP, and FN are the number of true positive, true negative, false positive, and false negative predictions, respectively. The MATLAB source code in this paper is freely available to the public upon request.

## Figures and Tables

**Table 1:** Running time for PCNV, Bayesian inference, FFP, AFKS, and Muscle methods. “∼”, unable to compute on laptop.

Method	HCV	HBV	Dengue	HPV	Bacteria
	(82)	(152)	(330)	(326)	(59)
PCNV	0.33s	0.27s	0.66s	0.78s	53.71s
Bayesian	1097s	263s	217,353s	217,512s	∼
inference					
FFP	11.11s	0.38s	49.40s	35.00s	larger than
	(k = 6)	(k = 5)	(k = 6)	(k = 6)	1 day (k = 11)
AFKS	70.21s	29.62s	429.87s	413.79s	larger than
	(k = 5)	(k = 4)	(k = 5)	(k = 5)	4 day (k = 9)
Muscle	753s	155s	3740s	4002s	∼

**Table 2:** Sensitivity (Sens), Specificity (Spec), and Accuracy (Acc) measures of classification are reported for the four virus datasets. For each dataset, the Ave. line displays average values for each measure.

	Nu-	Sens	Sens	Sens	Spec	Spec	Spec	Acc	Acc	Acc	
	Type	mber	PCNV	FFP	AFKS	PCNV	FFP	AFKS	PCNV	FFP	AFKS
			(%)	(%)	(%)	(%)	(%)	(%)	(%)	(%)	(%)
HCV (82)	type1	16	100	62.5	50.0	100	87.9	86.4	100	62.5	50.0
	type2	18	100	55.6	94.4	100	93.8	98.4	100	55.6	94.4
	type3	20	100	80.0	90.0	100	93.5	96.8	100	80.0	90.0
	type4	12	100	50.0	33.3	100	97.1	90.0	100	50.0	33.3
	type5	4	100	50.0	75.0	100	96.2	97.4	100	50.0	75.0
	type6	12	100	50.0	83.3	100	91.4	98.6	100	50.0	83.3
	Ave.		100	58.0	71.0	100	93.3	94.6	100	58.0	71.0
HBV (152)	A	20	100	100	100	100	100	99.2	100	100	100
	B	15	100	100	40.0	100	100	96.4	100	100	40.0
	C	20	100	100	70.0	100	100	96.2	100	100	70.0
	D	13	100	100	76.9	100	100	97.1	100	100	76.9
	E	30	100	100	90.0	100	100	97.5	100	100	90.0
	F	22	100	100	72.7	100	100	93.8	100	100	72.7
	G	17	100	100	94.1	100	100	99.3	100	100	94.1
	H	15	100	100	80.0	100	100	97.1	100	100	80.0
	Ave.		100	100	78.0	100	100	97.1	100	100	78.0
Dengue (330)	type1	72	100	100	76.4	100	100	93.4	100	100	76.4
	type2	75	100	100	73.3	100	100	93.3	100	100	73.3
	type3	83	100	100	78.3	100	100	92.7	100	100	78.3
	type4	100	100	100	87.0	100	100	93.0	100	100	87.0
	Ave.		100	100	78.8	100	100	93.1	100	100	78.8
HPV (326)	6	24	100	100	75.0	100	100	97.7	100	100	75.0
	11	17	100	100	100	100	100	99.7	100	100	100
	16	99	100	100	92.9	100	100	96.5	100	100	92.9
	18	19	100	100	94.7	100	100	100	100	100	94.7
	31	23	100	100	82.6	100	100	99.0	100	100	82.6
	33	22	100	100	86.4	100	100	99.7	100	100	86.4
	35	26	100	100	88.5	100	100	99.0	100	100	88.5
	45	12	100	100	83.3	100	100	99.7	100	100	83.3
	52	22	100	100	81.8	100	100	98.4	100	100	81.8
	53	14	100	100	85.7	100	100	98.7	100	100	85.7
	58	37	100	100	94.6	100	100	99.7	100	100	94.6
	66	11	100	100	90.9	100	100	99.7	100	100	90.9
	Ave.		100	100	88.0	100	100	99.0	100	100	88.0

**Table 3:** Summary of the datasets HCV, HBV, Dengue, HPV, and Bacteria. The length distribution of each dataset validates that PCNV can work with long sequences.

Dataset	Number	Min	Median	Mean	Max
		(bp)	(bp)	(bp)	(bp)
HCV	82	8957	9442	9427	9666
HBV	152	10161	10669	10606	10780
Dengue	330	10,161	10,669	10,606	10,780
HPV	326	7814	7,905	7895	8051
Bacteria	59	846,214	4,016,947	3,610,938	5,966,919

**Table 4:** The positional distribution of “ACTGGCAAT”.

Sequence	A	C	T	G	G	C	A	A	T
Position	1	2	3	4	5	6	7	8	9
$U_{A}\left(i\right)$	$\frac{1}{6}$	$\frac{1}{6}$	$\frac{1}{6}$	$\frac{1}{6}$	$\frac{1}{6}$	$\frac{1}{6}$	$\frac{7}{1}$	$\frac{8}{2}$	$\frac{8}{2}$
$U_{C}\left(i\right)$	0	$\frac{2}{4}$	$\frac{2}{4}$	$\frac{2}{4}$	$\frac{2}{4}$	$\frac{6}{4}$	$\frac{6}{4}$	$\frac{6}{4}$	$\frac{6}{4}$
$U_{T}\left(i\right)$	0	0	$\frac{3}{6}$	$\frac{3}{6}$	$\frac{3}{6}$	$\frac{3}{6}$	$\frac{3}{6}$	$\frac{3}{6}$	$\frac{9}{1}$
$U_{G}\left(i\right)$	0	0	0	$\frac{4}{1}$	$\frac{5}{5}$	$\frac{5}{5}$	$\frac{5}{5}$	$\frac{5}{5}$	$\frac{5}{5}$

## Supplementary Materials

The information for all five datasets is shown in the Supplementary_File. The following are available at https://www.mdpi.com/1422-0067/21/11/3859/s1.

## Abbreviations

The following abbreviations are used in this manuscript:

AFKS	Alignment-Free-Kmer-Statistics
DENV	Dengue virus
HCV	Hepatitis C virus
HBV	Hepatitis B virus
HMM	hidden Markov model
HPV	Human papillomavirus
PCNV	Positional Correlation Natural Vector
NV	Natural Vector
ViPR	Virus Pathogen Database and Analysis Resource
FFP	Feature Frequency Profiles
