# Supplementary material for: Positional Correlation Natural Vector: A Novel Method for Genome Comparison
Source: Int J Mol Sci. 2020 May 29;21(11):3859. doi: 10.3390/ijms21113859 (PMC7312176; doi:10.3390/ijms21113859)
Supplement: Supplementary file 1 [file ijms-21-03859-s001.zip › ijms-785084 -for publication-supplementary/Figures.docx]

**Supplementary Data for**

**Positional correlation natural vector: a novel method**

**for genome comparison**

Lily He^1^, Rui Dong^1^, Rong Lucy He^2^ and Stephen S.-T. Yau^1,^*

^1^ Department of Mathematical Sciences, Tsinghua University, Beijing, 100084, China

^2^ Department of Biological Sciences, Chicago State University, Chicago, Illinois, IL 60628, USA

* To whom correspondence should be addressed. Tel: +86-10-62787874; Fax: +86-10-62798033; Email: [yau@uic.edu](mailto:yau@uic.edu)


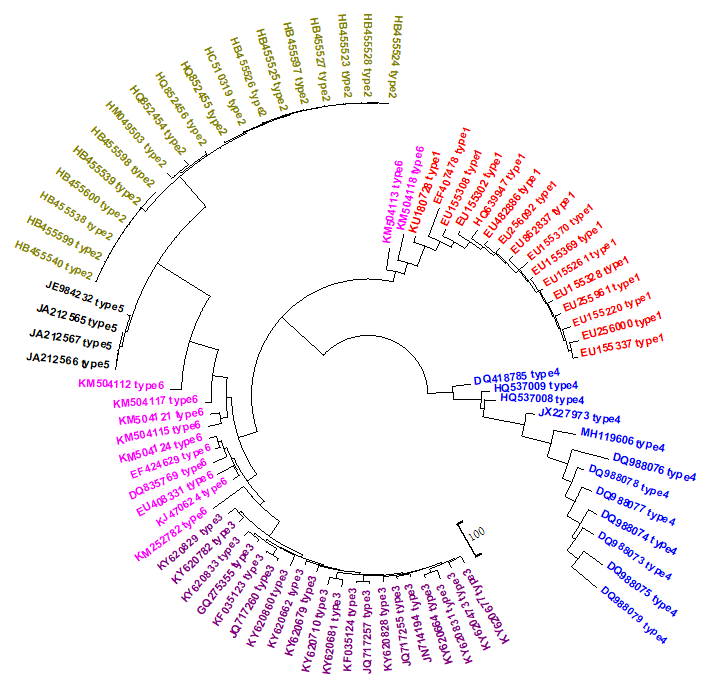
Figure S1: The Neighbor-Joining phylogenetic tree of 82 HCV genome sequences based on the MEV method


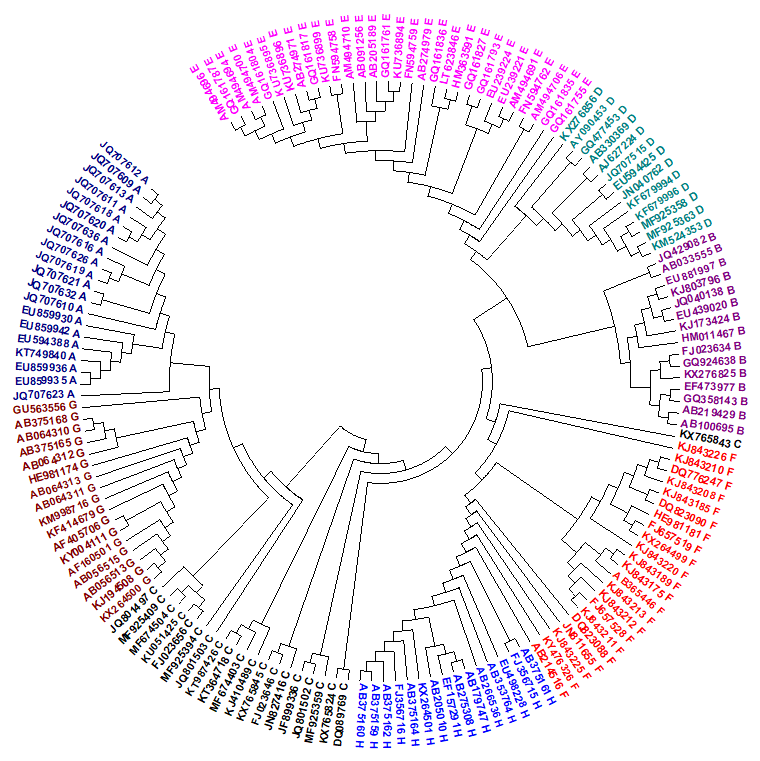


Figure S2: The Neighbor-Joining phylogenetic tree of 152 HBV genome sequences

based on the MEV method


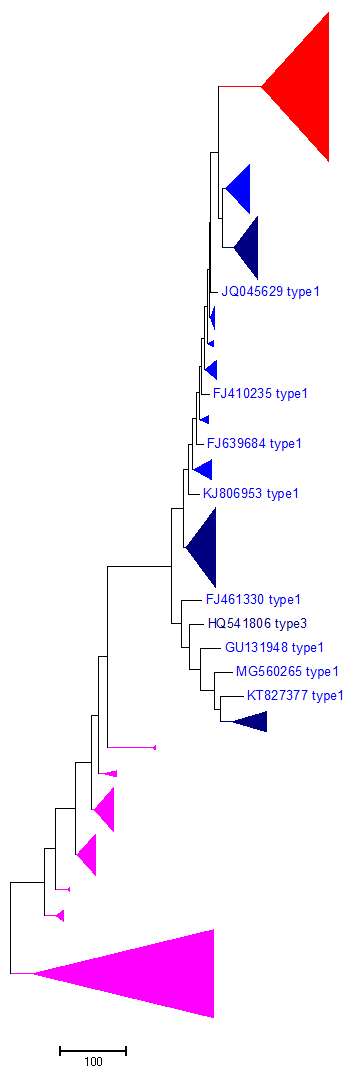


Figure S3: The Neighbor-Joining phylogenetic tree of 330 dengue genome sequences based on the MEV method


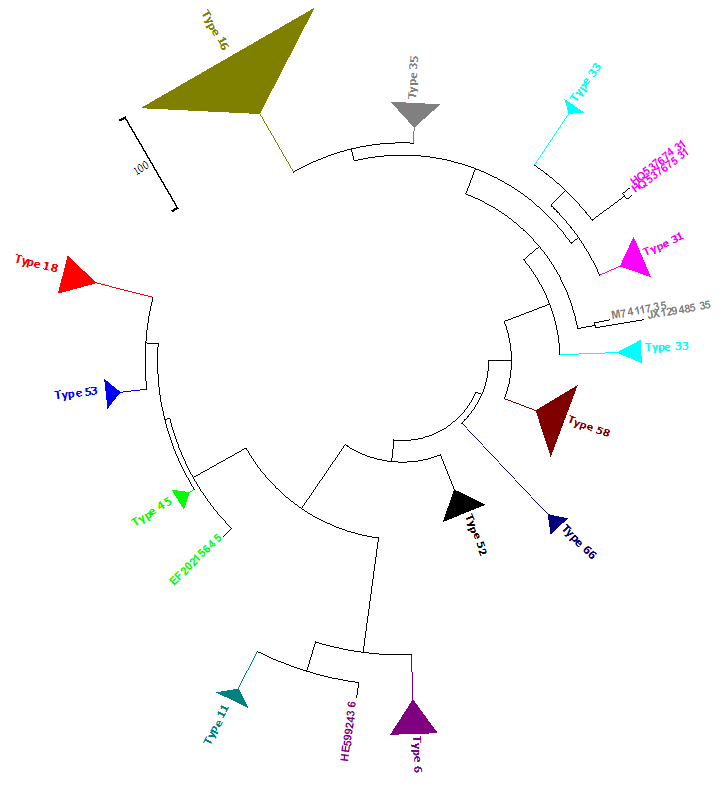


Figure S4: The Neighbor-Joining phylogenetic tree of 326 HPV genome sequences based on the MEV method


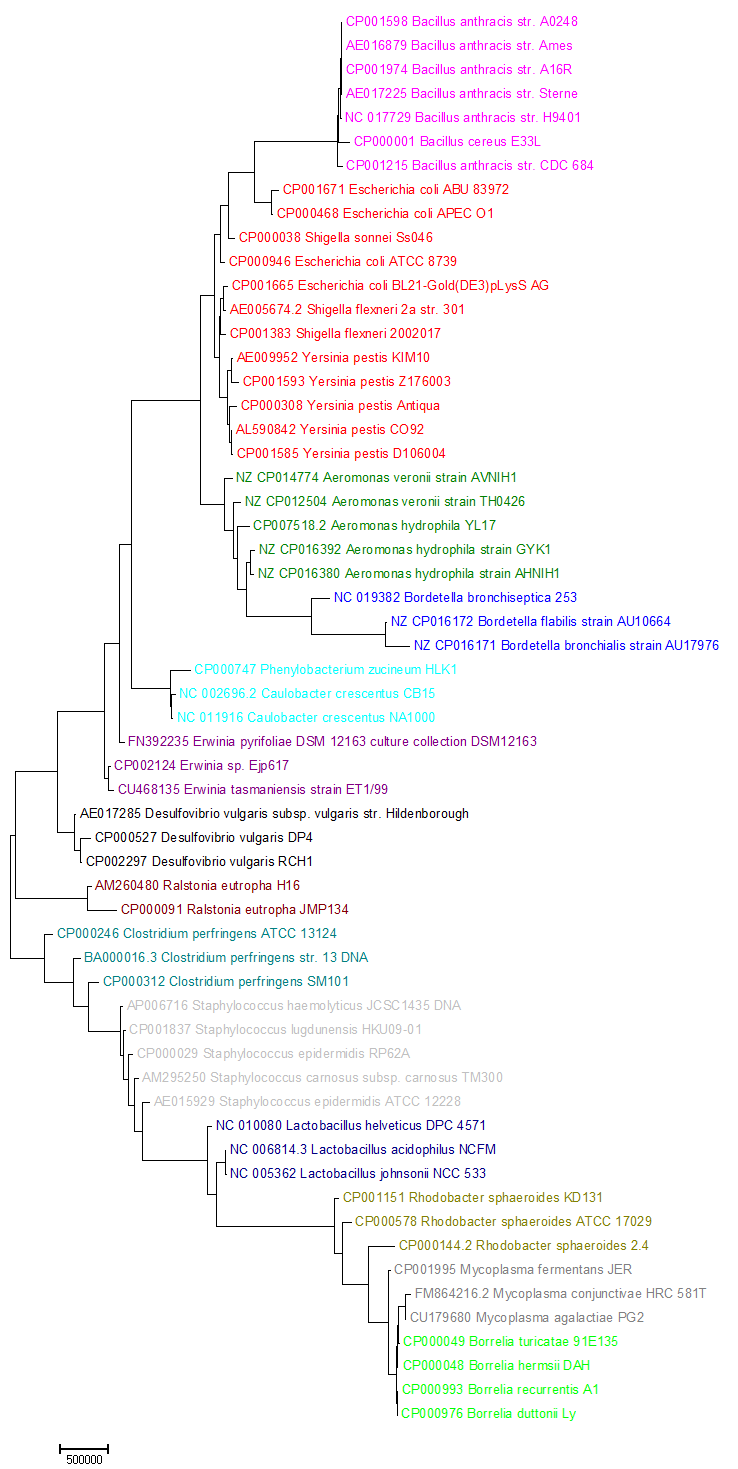


Figure S5: The Neighbor-Joining phylogenetic tree of 59 bacteria genome sequences based on the MEV method
